# Assessing the Impact of a Positive Psychology School‐Based Psychoeducation on the Mental Health of Adolescents Affected by the 2023 Earthquake in Türkiye

**DOI:** 10.1002/jad.70153

**Published:** 2026-04-08

**Authors:** Veysel Kaplan, Mehmet Emin Düken, Abanoub Riad, Muhammad Alkasaby

**Affiliations:** ^1^ Nursing Department, Faculty of Health Sciences Harran University Şanlıurfa Turkey; ^2^ Department of Public Health, Faculty of Medicine Masaryk University Brno Czech Republic; ^3^ Health in Humanitarian Crises Centre, London School of Hygiene & Tropical Medicine London UK

**Keywords:** adolescents, coping, earthquake, future expectation, mental health, positive psychology, psychoeducation

## Abstract

**Introduction:**

The purpose of this study is to examine the effectiveness and acceptability of a positive psychology‐based psychoeducation program on the psychological states, future expectations, and coping styles of adolescents affected by the 2023 earthquake in Türkiye.

**Methods:**

A single‐group, pretest‐posttest design was adopted. A four‐session psychoeducation program was delivered to 74 students in one of the high schools in a village affected by the earthquake. The Brief Symptom Inventory (BSI), Future Expectations Scale for Adolescents (FESA), and Coping Scale for Adolescents (KIDCOPE) were used to assess the intervention outcomes. Additionally, interviews were used to assess the intervention's acceptability and perceived impact.

**Results:**

There was a statistically significant decrease in the total BSI score and all sub‐dimensions after the intervention (*p* < 0.001). While there was no significant difference between the KIDCOPE general scores (*p* = 0.599), significant improvements were recorded in the active coping (*p* = 0.031) and negative coping (*p* = 0.003) sub‐dimensions. Additionally, there was an increase in FESA scores, with a statistically significant difference, especially in the marriage and family sub‐dimension. Students perceived the interventions as impactful, interactive, timely, and culturally relevant. They suggested including their families in the intervention and reducing the group size to allow more interaction.

**Conclusion:**

Mental health interventions targeting children and adolescents should not only focus on reducing negative symptoms but also on nurturing positive mental health traits. Additionally, families should be included in the interventions as they play a vital role in adolescents' emotional and social well‐being.

## 1 Introduction

Earthquakes can lead to widespread destruction and disrupt the normal flow of life, often being associated with significant challenges to physical and psychological well‐being (Alipour and Ahmadi 2020; Cihanoğlu et al. 2024). Türkiye, a country with 42% of its surface area in the first‐degree earthquake zone and a large majority of its population living in earthquake‐risk regions, has experienced numerous earthquakes of various magnitudes throughout its history (Yanardağ 2024). Most recently, two of the strongest earthquakes of recent times occurred in Türkiye on 6 February 2023 in Pazarcık District (7.8 Mw) and Elbistan District (7.7 Mw) of Kahramanmaraş province (Afet ve Acil Durum Yönetimi Başkanlığı AFAD 2023). These earthquakes were the second‐ and third‐strongest in Türkiye (İstanbul Teknik Üniversitesi İTÜ 2023). They directly affected 11 provinces, in addition to other countries such as Syria, and caused significant destruction and loss of lives and livelihoods. In Türkiye, around 50,000 people were killed, and more than 100,000 were injured (Afet ve Acil Durum Yönetimi Başkanlığı AFAD 2023). In response, container and tent cities were set up for survivors, with a significant number of people either relocating or being evacuated. Records indicate that by February 24, 2023, over 528,000 people had been evacuated from the affected regions (Afet ve Acil Durum Yönetimi Başkanlığı AFAD 2023).

While earthquakes and their aftermath can affect individuals across all ages, socioeconomic and cultural backgrounds, some studies suggest that children and adolescents may be particularly vulnerable to psychological distress in post‐disaster settings. For instance, qualitative research conducted among teenagers following the 2010–2011 New Zealand earthquakes underscores that adolescents' recovery trajectories and psychosocial needs differ markedly from those of adults and points to both risk and resilience factors specific to their developmental stage (Pine et al. 2015). The psychological impact of earthquakes on adolescents has been linked to the ongoing physical, cognitive, and social development characterizing this life stage, and their coping mechanisms for stressful/challenging situations might not be sufficiently developed (Can Gür 2024; Rubens et al. 2018). These developmental characteristics may increase adolescents' susceptibility to more pronounced psychological effects following traumatic events such as earthquakes (Kaplan et al. 2024). Many studies indicate that experiences such as the loss of family members, food insecurity, and damage to homes or schools may represent substantial threats to adolescents' psychosocial and existential resources (Hobfoll 1989; Hobfoll and Schumm 2009; Yanardağ 2024).

Within the disaster psychology literature, Conservation of Resources (COR) theory provides a useful framework for understanding why adolescents may be particularly vulnerable following large‐scale crises such as earthquakes, as sudden losses of safety, routines, social support, and future‐related resources may increase the risk of psychological distress rather than deterministically leading to psychopathology (Can Gür 2024; Hobfoll et al. 1990; Hobfoll and Schumm 2009). Some studies have consistently reported elevated levels of psychological distress among some children and adolescents after earthquakes, while also highlighting substantial heterogeneity in post‐disaster responses (Gökler Danışman and Okay 2017; Karabulut and Bekler 2019). For example, Liang et al. (2019), in a 4‐year longitudinal study following the Wenchuan earthquake, identified three distinct PTSD symptom trajectories among children—resilient, relapsing, and recovery—demonstrating that long‐term psychological outcomes vary rather than follow a uniform pattern and are shaped by individual, contextual, and environmental factors.

From a COR perspective, crises may also be associated with shifts in coping processes, with disaster‐exposed individuals showing greater reliance on less adaptive coping strategies such as avoidance or self‐blame, which have been linked to poorer psychological functioning (Hobfoll 1989; Xu and He 2012). Moreover, adolescents who experience psychological distress and difficulties in coping after earthquakes have been found to report less positive expectations regarding the future (Saupe et al. 2019; Yilmaz et al. 2005). Despite growing evidence documenting post‐disaster psychological risks, research examining applied psychosocial interventions for children and adolescents in post‐disaster contexts remains limited, particularly regarding school‐based and preventive approaches (Pine et al. 2015). This gap underscores the need for exploratory, applied studies that evaluate interventions targeting not only symptom reduction but also coping strategies and future orientation among adolescents affected by earthquakes. In response to these gaps, various intervention strategies have been developed to support adolescents' mental health in post‐disaster contexts, including psychotherapeutic techniques such as relaxation, affect modulation, coping, and exposure (Pfefferbaum et al. 2014), as well as broader intervention approaches such as digital interventions, trauma‐focused CBT, and resilience‐building workshops (Cohen and Mannarino 2017; Masten and Reed 2002; Ruggiero et al. 2015). In addition, many other interventions focused only on specific symptoms experienced by the adolescents and on acute problem‐solving, but have not addressed the long‐term impact of earthquakes (Chen et al. 2014; Lai et al. 2016; Sugiyama et al. 2020). Against this backdrop, positive psychology focuses on developing human strengths and capacities rather than focusing solely on symptoms or deficits (Seligman and Csikszentmihalyi 2000). In this process, the main focus is on positive processes such as being in the moment, feeling optimism and hope for the future, taking responsibility, showing love to others, and being courageous towards innovations and actions aimed at benefiting society (Seligman 2002). Accordingly, the present study aims to examine the effects of a brief, school‐based psychoeducation program grounded in positive psychology on adolescents' psychological well‐being, coping strategies, and future expectations in a post‐earthquake context. In this study, we hypothesized that a positive psychology‐based psychoeducation program would positively affect the mental states, coping styles, and future expectations of adolescents affected by the earthquake.

## 2 Methods

### 2.1 Study Design

We employed a one‐group pretest‐posttest design to evaluate the effectiveness of a positive psychology‐based psychoeducation program in improving psychological symptoms, future expectations and coping skills of adolescents in a village school affected by the earthquake. Additionally, we conducted interviews to assess acceptability and feasibility and collected participants' feedback to help us further improve the interventions.

### 2.2 Participants

#### 2.2.1 Recruitment Process

Firstly, we visited village schools in areas affected by the earthquake near the city of Şanlıurfa, which are easily accessible to the research team. We explained the purpose of the study and the content of the intervention to the school administration. Among the schools we visited, three schools were eligible based on the following criteria:

- The school has a seminar hall, sound system and trees in the garden (these are the equipment needed for the implementation of the planned intervention),
- The school building is not physically damaged due to the earthquake, to ensure the safety of students and the research team during the implementation, and
- The school should be able to continue education after the earthquake.

We used a Research Randomiser program to select one of the schools that met the intervention criteria. The school we chose had 678 students enrolled on grades 9–12, but only 422 students were continuing their education. Since the school psychological guidance services have recently conducted psychoeducational activities for 9th, 10th, and 11th‐grade students, our intervention was implemented only with 12th‐grade students. In addition to the earthquake's impact, 12th‐grade students are preparing for university exams, which adds another layer of stress and underscores the need for psychoeducation. We implemented the intervention with 74 students who met the following inclusion criteria:

- Being enrolled in the 12th grade,
- Willing to participate in the study,
- Currently lives in the province affected by the earthquake,
- Continuing their education in the school,
- Not having participated in any psychoeducational program in the last year.
- Not diagnosed with mental disorders in the past,
- Not having other issues that may prevent them from communicating clearly during the data collection and psychoeducation (e.g., hearing problems).

### 2.3 Data Collection

#### 2.3.1 Quantitative Data

The surveys were administered by the researchers, with assistance from school staff, in a classroom setting at the participating village school. Prior to data collection, participants were informed about the purpose of the study and voluntary participation was emphasized. The questionnaires were completed individually during school hours (08:00–15:00) under the supervision of the researchers. For the purpose of this study, we created a survey that consists of the following:

##### 2.3.1.1 Personal Information Form

The form includes questions about the parents' age, education level, and working status, as well as the number of siblings.

##### 2.3.1.2 Brief Symptom Inventory (BSI)

The scale was developed by Derogatis (1992) and validated in Turkish by Şahin and Durak (1994). The scale, which assesses mental symptoms (anxiety, depression, negative self‐concept, somatization and hostility), is a 4‐point Likert‐type scale and consists of 53 items. The score obtained from the scale ranges from 0 to 212, with higher scores indicating the frequency of symptoms in the individual. Cronbach's alpha internal consistency coefficients obtained from the subscales range between 0.55 and 0.86 (Şahin and Durak 1994).

##### 2.3.1.3 Future Expectations Scale for Adolescents (FESA)

The scale was developed by McWhirter and McWhirter (2008) and adapted into Turkish by Tuncer (2011). It covers four areas: Work and Education, Marriage and Family, Religion and Society, and Health and Life. The scale consists of 25 items in total and is a 7‐point Likert‐type scale. With a score range of 25–175, high scores indicate positive expectations for the future. Cronbach's alpha internal consistency coefficients obtained from the subscales range between 0.65 and 0.78 (Tuncer 2011).

##### 2.3.1.4 Coping Scale for Adolescents (KIDCOPE)

The Coping Scale for Adolescents (KIDCOPE) was developed by Spirito et al. (1988), and its validity and reliability in Turkish were examined by Bedel et al. (2014). The scale consists of 11 items rated on a 4‐point Likert‐type scale and includes three subdimensions: Active Coping, Avoidant Coping, and Negative Coping. Higher scores indicate greater use of the corresponding coping strategy. Cronbach's alpha internal consistency coefficients obtained from the subscales range between 0.65 and 0.72 (Bedel et al. 2014).

#### 2.3.2 Qualitative Data

We have interviewed eight students to understand their perceptions of the intervention's impact and gather feedback on how it could be improved. Eight students were purposively selected from among the participants who completed the intervention to provide in‐depth feedback on their experiences with the program. Interviews were conducted after the completion of the psychoeducational program. The qualitative component aimed to provide preliminary insights into participants' experiences with the intervention rather than to develop a comprehensive theoretical model.

The interviews were conducted by the first author (the positive psychology practitioner in the study) in the guidance and psychological counseling teacher's office at the school. Each interview lasted approximately 25–40 min. With the participants' consent, the interviews were audio‐recorded and later transcribed verbatim for analysis. The interview questions covered the following topics:

1. Aspects found useful or inadequate in the intervention,
2. Alignment of the intervention with the needs and goals of the participants,
3. Changes experienced as a result of the intervention,
4. Cultural appropriateness of the intervention,
5. Suggestions for improving the intervention.

### 2.4 Implementation Process

Ahead of delivering the intervention, meetings were organized with the school administration and psychological guidance services to discuss logistical arrangements, including the timing of the intervention and the organization of the hall. The study was implemented over a 6‐week period. In the first week, students were informed about the research process and the content of the psychoeducation sessions. Information was presented in age‐appropriate, clear language, and sufficient time was allocated to ensure that students fully understood the purpose, procedures, potential benefits, and the study's voluntary nature. Students were encouraged to ask questions, and clarifications were provided when needed. Written informed consent was obtained from parents or legal guardians, and assent was obtained from all participating students. Participants were informed that they could withdraw from the study at any time without any academic or personal consequences. Following this, pre‐test measures were administered. Additionally, in the first session, participants were provided with small educational materials (e.g., a notebook and pen) to support note‐taking. These items were provided to all participants and were not contingent on attendance or completion of the study. In the 2nd, 3rd, 4th, and 5th weeks, four psychoeducation sessions were delivered. In the 6th week, post‐test measures were administered, qualitative interviews were conducted, and the study was formally concluded.

### 2.5 Intervention

This psychoeducational intervention was implemented as a pilot and exploratory program to assess feasibility, acceptability, and short‐term psychosocial outcomes in a real‐world school setting following a major disaster. It was designed and delivered by the first author, drawing on core principles of positive psychology (Seligman and Csikszentmihalyi 2000; Seligman 2002) and informed by disaster psychology and trauma‐informed care. The program was developed to address psychosocial resource depletion and emotional distress following the earthquake, with a specific focus on enhancing strengths, adaptive coping, hope, and interpersonal relationships among adolescents. While designing the program, we took into consideration the cultural and contextual characteristics of adolescents living in rural, earthquake‐affected settings in Türkiye.

The intervention consisted of 4 weekly sessions, each lasting approximately 60 min. Table 1 summarizes the objectives and content of each session, and additional details are provided in the Supporting Information S1. Sessions were conducted in the school seminar hall or garden, depending on the planned activities and session objectives. The program began with a strengths‐based session to foster emotional safety and self‐efficacy, then gradually introduced stress‐coping strategies and self‐reflection. Coping strategies were framed within everyday experiences, avoiding intensive trauma exposure. The final session focused on positive relationships and social support to promote emotional containment and a sense of connection at the conclusion of the program.

**Table 1:** Intervention details.

Session theme	Sessions details	Session activities
1. Character Strengths and Virtues	The session focuses on identifying positive characteristics/virtues about oneself. It started with introduction and explaination of the program. Information was given to raise awareness about the strong, positive and open to development characteristics that exist in every individua.	Q&A, case study, brainstorming, positive interpretation, role‐play
2. Optimism and Hope for the Future	The session focuses on being optimistic in one's daily life and having positive dreams for the future Students were given information and examples on how to have positive feelings and optimism in their daily lives and positive dreams for the future.	Q&A, case study, reframing, positive interpretation, dreaming, setting goals.
3. Self‐Understanding and Struggle	The session focuses on enabling participants to be more understanding of themselves and to increase their self‐awareness. In addition, students learned about some methods that can be used to cope with stress	Q&A, brainstorming, physical exercise, empty chair.
4. Positive Relationships	The session focuses on establishing more effective relationships with others. Topics such as the effects of our relationships on us and those around us, healthy‐unhealthy relationships, you‐me language and effective communication skills were discussed.	Q&A, brainstorming, case study, role‐play.

### 2.6 Data Analyses

As part of quantitative data analysis, descriptive statistics (mean ± SD, median, minimum, maximum, number (%), etc.) were first performed. The pre‐test/post‐test comparison for the group was performed using a paired *t*‐test. The results were evaluated using a 95% confidence interval and a *p*‐value < 0.05.

Qualitative data were analyzed using thematic analysis following the approach described by Braun and Clarke (2006) and Braun and Clarke (2021). Because the interviews were conducted to evaluate participants' experiences with the psychoeducational intervention, a primarily deductive approach was adopted. The coding process was informed by the topics included in the semi‐structured interview guide and the program evaluation framework. The analysis involved several stages. First, the interview transcripts were read multiple times to achieve familiarity with the data. Next, meaningful data segments were coded in relation to the evaluation topics addressed in the interviews. These codes were then organized into broader themes that reflected participants' perceptions of the intervention. The themes were reviewed and refined through iterative comparison with the coded data and the full dataset. Coding and theme development were conducted by the authors through iterative discussions to ensure consistency in data interpretation. Verbatim quotations from participants were used to illustrate each theme.

### 2.7 Reflexivity

Reflexivity was actively considered throughout the qualitative research process. The interviews, data analysis, and interpretation were conducted by authors (Veysel Kaplan and Muhammed Alkasabay), who have academic training and professional experience in adolescent mental health, psychosocial interventions, and trauma‐informed care. While this background facilitated sensitivity to participants' experiences, it may also have shaped initial assumptions during data interpretation. To address this, the researchers engaged in iterative data immersion, reflexive memo writing, and ongoing critical reflection to examine how personal assumptions and prior professional experiences might influence data interpretation.

### 2.8 Ethical Considerations

Ethical committee (Protocol Number: E‐76244175‐050.04.04‐224439/24.04.2023) and institutional permissions (Protocol Number: E‐98910266‐199‐74254126/12.04.2023) for the study were obtained during the planning phase. The study information sheet and consent form were shared with the adolescents, their parents and the school administration before data collection. The form contained information about the purpose and duration of the study, and participants' rights. Participants were informed that they could withdraw from the study at any time and that all information would be kept confidential and used only for the stated research purposes.

## 3 Results

### 3.1 Quantitative Results

A total of 74 students received the intervention. Participants' sociodemographic information is summarized in Table 2.

**Table 2:** Some sociodemographic characteristics of the participants.

Characteristics	Categories	*n*	%
Sex	Male	23	31
	Female	51	69
Mother education level	Illiterate	39	53
	Literate	4	5
	Primary school	30	41
	High school	1	1
Father education level	Illiterate	7	9
	Literate	3	4
	Primary school	52	70
	High school	12	16
Mother occupation	Housewife	74	100
Father occupation	Farmer	41	55.4
	Freelance (Tradesman)	8	10.8
	Worker	18	24.3
	Driver	4	5.4
	Unemployed	3	4.1

	Mean ± SD	Median (Min‐Max)
Age	16.58 ± 1.11	17 (15–19)
Number of siblings	6.12 ± 2.49	6 (1–14)

Table 3 shows the differences in mean scores on the BSI and its subscales before and after the intervention. The BSI mean scores after the intervention are significantly lower than the mean scores before the intervention, with a statistically significant difference (*p* < 0.001). Similarly, statistically significant differences are evident across all BSI subscales, indicating that our intervention contributed to reductions in psychological symptoms among adolescents.

**Table 3:** Mean scores of the BSI and subscales before and after the intervention.

Scale	Pre‐Test		Post‐Test		Difference		
	Mean ± SD	Median (Min–Max)	Mean ± SD	Median (Min–Max)	Mean ± SD	Test Statistic	*p*
BSI	126.41 ± 27.22	126 (89–195)	56.62 ± 23.45	62 (0–89)	69.78 ± 22.4	18.946	***p*** < **0.001**
Anxiety	29.08 ± 10.22	30 (11–50)	12.3 ± 6.59	12 (0–24)	16.78 ± 9.04	11.289	***p*** < **0.001**
Depression	32.86 ± 7.74	33 (20–47)	14.38 ± 8.47	16 (0–38)	18.49 ± 8.75	12.844	***p*** < **0.001**
Negative self	28.35 ± 7.04	28 (15–44)	11.51 ± 6.67	12 (0–27)	16.84 ± 9.36	10.937	***p*** < **0.001**
Somatisation	18.68 ± 8.2	18 (4–35)	8.14 ± 5.09	8 (0–20)	10.54 ± 9.03	7.103	***p*** < **0.001**
Hostility	17.43 ± 4.72	17 (10–26)	10.3 ± 5.96	11 (0–25)	7.14 ± 6.84	6.345	***p*** < **0.001**

As shown in Table 4, the total KIDCOPE score did not show a statistically significant change following the intervention (*p* = 0.607). However, a statistically significant increase in Active Coping strategies (*p* = 0.029) and a significant decrease in Negative Coping strategies (*p* = 0.001) were observed, indicating a shift toward more adaptive coping patterns after the psychoeducational program. No significant change was found in Avoidant Coping strategies (*p* = 0.157). These findings suggest that, although the intervention was effective in strengthening constructive coping behaviors and reducing maladaptive responses, avoidant coping strategies may be more resistant to change and may require more targeted or prolonged intervention components.

**Table 4:** Mean scores of the KIDCOPE and subscales before and after the intervention.

Scale	Pre‐Test		Post‐Test		Difference		
	Mean ± SD	Median (Min–Max)	Mean ± SD	Median (Min–Max)	Mean ± SD	Test Statistic	*p*
KIDCOPE	26.16 ± 3.9	26 (15–36)	25.7 ± 3.6	26 (17–35)	0.46 ± 5.39	0.519	0.607
Active Coping	9.65 ± 2.6	10 (4–14)	10.97 ± 2.6	11 (5–16)	−1.32 ± 3.54	−2.278	**0.029**
Avoidant Coping	10.27 ± 2	10 (6–16)	9.62 ± 1.7	10 (6–13)	0.65 ± 2.73	1.445	0.157
Negative Coping	6.24 ± 1.7	6 (3–9)	5.11 ± 1.4	5 (3–8)	1.14 ± 1.96	3.522	**0.001**

As shown in Table 5, a statistically significant improvement was observed only on the Marriage and Family subscale of the FESA following the intervention (*p* = 0.014), indicating more positive future expectations in family and marital domains. Although increases were also observed in the total FESA score and other subscales (work and education, church and community, and health), these changes did not reach statistical significance.

**Table 5:** Mean scores of the FESA and subscales before and after the intervention.

Scale	Pre‐Test		Post‐Test		Difference		
	Mean ± SD	Median (Min‐Max)	Mean ± SD	Median (Min‐Max)	Mean ± SD	Test Statis.	*p*
FESA	116.76 ± 33	121 (28–175)	132.27 ± 28	140 (49–175)	−15.51 ± 47.79	−1.975	**0.049**
Work and education	55.97 ± 17	57 (11–77)	60.92 ± 16.2	68 (13–77)	−4.95 ± 25	−1.203	0.237
Marriage and family	28.16 ± 12.6	27 (7–49)	35.49 ± 9.5	37 (7–49)	−7.32 ± 17.21	−2.589	**0.014**
Church and community	14.54 ± 5.2	16 (3–21)	15.78 ± 4.8	17 (5–21)	−1.24 ± 7.49	−1.009	0.320
Health	18.08 ± 7	18 (4–28)	20.08 ± 6.1	22 (4–28)	−2.00 ± 8.90	−1.367	0.180

In conclusion, mean scores on the BSI, FESA, and KIDCOPE scales, as well as their subscales, differed between the pre‐ and post‐intervention assessments. However, given the absence of a control group, these changes should not be interpreted as definitive causal effects of the intervention, but rather as short‐term improvements observed within the intervention group.

### 3.2 Qualitative Findings

The analysis resulted in three overarching themes reflecting participants' experiences with the intervention, which are summarized in Table 6.

**Table 6:** Summary of the qualitative findings.

Theme	Subtheme(s)
Characteristics of the intervention	Sessions are interactive Relevance to the context Timing of the intervention (before the exams, after the earthquake)
Perceived benefits	Talking to friends and realizing that they are not alone in their feelings Discovering/realizing positive aspects of their personalities Improving future expectations
Suggested Improvements	Reducing the number of participants in each session Delivering the intervention to families

#### 3.2.1 Intervention Characteristics

Students generally provided positive feedback regarding the characteristics of the intervention. The timing of the intervention was particularly appreciated, as it coincided with the university exam preparation period and followed the earthquake. This timing was seen as providing much‐needed support during a challenging time. The format of the intervention, which was interactive and included activities outside of a traditional lecture setting, was also viewed favorably, making it *“very fun.”* However, one significant characteristic mentioned as a negative aspect was the group size, with students noting that it was *“too crowded.”*

The psychoeducation program was not just like a lecture/lesson. It was very fun. It was not just in the conference hall. We also did activities in the garden. I was very excited during the psychoeducation. But it was very crowded.

—Participant 5 (17 years old, Male)

The intervention activities, such as the “famous people activity” and the “tree activity,” were highlighted as particularly engaging and beneficial, providing new perspectives and making daily tasks feel more enjoyable. Students also felt that several aspects of the intervention were highly relevant to their context and personal experiences. For example, the examples used, including those about successful famous people like Nobel Prize winner Aziz Sancar, were relatable and encouraging, demonstrating that success is possible regardless of background.

It was very good to get to know all the successful, famous people shown during the psychoeducation and to see that they were like us once. For example, Nobel Prize winner Aziz Sancar also studied in a village school, like us. Seeing this encouraged me. It was very good that the psychoeducation told us about ourselves. It was very suitable for my life.

—Participant 3 (17 years old, Female)

#### 3.2.2 Perceived Benefit of the Intervention

The perceived benefits of the intervention were wide‐ranging, impacting students' emotional state, self‐perception, and future planning. Many students reported feeling happier and better after the intervention. The intervention helped address existing fears and anxieties, with one student sharing,

I have started to feel better after the psychoeducation. I have had fears for a long time. This psychoeducation was very good. It was great for my needs.

—Participant 3 (17 years old, Female)

Students also felt less alone in their struggles after discussing common fears, such as the fear of earthquakes, with their peers.

I am always afraid that an earthquake will happen again. We didn't talk about earthquakes with our friends. Everyone was afraid of earthquakes, like me. When we talked about this issue together, I realized that I was not alone. There is nothing negative about it.

—Participant 8 (17 years old, Female)

A significant benefit was the shift in perspective regarding personal characteristics and success. Students discovered their positive characteristics and learned that success is tied to personal desires, not just to family expectations. The intervention also encouraged self‐reflection and self‐awareness, with one student starting a diary to better understand themselves.

Thanks to the activities we did during the psychoeducation, I discovered that I have some positive characteristics. And my friends love these characteristics of mine.

—Participant 7 (17 years old, Male)

The intervention also had a positive impact on interpersonal relationships and future planning. Students reported improved communication with friends and a greater desire to support each other. The intervention motivated students to think about their future and make detailed plans, with one student expressing a strengthened desire to pursue their dreams, stating,

In the future, I want to do different things instead of working in the farm like my family.

—Participant 3 (17 years old, Female)

#### 3.2.3 Suggested Improvements

Students offered several suggestions for improving the intervention for future participants. A prominent suggestion was the need for similar psychoeducation for families. Students felt that their families could also benefit from learning to cope with challenges and support each other, particularly in the aftermath of the earthquake.

A similar psychoeducation needs to be done for our families. Instead of blaming themselves or others.

—Participant 3 (17 years old, Female)

Another improvement suggested was to reduce the number of students participating in each session to give an opportunity for more students to participate in the discussion.

It was very good that the event was held during our exam preparation period and at a time when we needed it the most after the earthquake. But our group was too crowded for the events.

—Participant 1 (17 years old, Male)

Student feedback highlights the intervention's positive effects on emotional well‐being, self‐awareness, and future aspirations, while suggesting improvements such as family involvement and smaller group sizes to enhance interaction and impact.

## 4 Discussion

The present study aimed to measure changes in the psychological symptoms, future expectations, and coping styles of adolescents affected by the earthquake following a positive psychology‐based intervention. Results showed that our intervention positively impacted adolescents' psychological well‐being. Additionally, qualitative findings identified aspects of the intervention that students found useful and suggested ways to improve it.

Results indicated a significant decrease in students' psychological symptoms after the intervention. In fact, the decrease occurred across all symptoms measured by the BSI; anxiety, depression, negative self‐concept, somatization and hostility. When examining the literature, interventional studies used several approaches to address the psychological problems (such as depression, anxiety, PTSD, grief, etc.) among adolescents affected by earthquakes and other traumatic events. Debriefing, psychological first aid, Cognitive Behavioral Therapy (CBT), Eye Movement Desensitization and Reprocessing (EMDR), and trauma‐focused approaches were commonly used, and significant positive changes similar to our results were obtained (Cain et al. 2010; Catani et al. 2009; Cohen and Mannarino 2017; İme 2024; Pfefferbaum et al. 2015; Salloum et al. 2009; Scheeringa et al. 2011; Trentini et al. 2018). However, these are generally problem‐focused, in contrast to positive psychology, which suggests that individuals should be identified and developed in terms of their positive aspects rather than their deficiencies (Seligman 2002). Positive Psychology‐based approaches build on people's resources, capacities, and strengths to improve well‐being and quality of life and achieve lifelong development (Boniwell 2012; Seligman and Csikszentmihalyi 2000). Accordingly, instead of focusing on psychological issues, our psychoeducation program raised awareness of the strengths and positive characteristics in every individual, which we think has contributed to the improvement of psychological symptoms.

Another change measured after the psychoeducation program is th use of coping strategies. There was an increase in the use of active coping among participants and a decrease in negative coping. The observed increase in active coping and reduction in negative coping should be interpreted cautiously, given that no significant change was observed in avoidant coping strategies. This pattern suggests that the intervention may have supported the development of more adaptive coping responses without substantially altering avoidance‐based strategies. According to Lazarus and Folkman (1984), people are not passive beings, and their reactions and coping strategies change depending on how they evaluate themselves and their experiences. Similarly, literature showed that including topics such as situation assessment, relaxation techniques and/or social relationships in psychoeducation interventions implemented after earthquakes and other crises was very important in developing effective coping skills (Alipour and Ahmadi 2020; Demirci et al. 2024; Fukui and Toyoshima 2008; İme 2024; Salimpoor et al. 2011; Streeter et al. 2012).

The last parameter measured after our psychoeducation intervention was adolescents' future expectations. We found a significant change in the total score of this parameter, and only for the marriage and family dimension. This change could be attributed to the psychoeducation program contents that focused on optimism, hope for a better future, and realizing one's strengths, which may help achieve a better future, which are concepts rooted in the basic principles of positive psychology (Hellman et al. 2018; Kun et al. 2017; Seligman et al. 2009). Several studies have shown that positive psychology interventions are an important tool for supporting “Hope” for the future among adolescents (Baourda et al. 2024; Ekinci and YILMAZ 2022; Marques et al. 2011; Teodorczuk et al. 2020).

The observed changes in psychological symptoms, coping styles, and future‐oriented expectations should be interpreted within the methodological limitations of the study design. Qualitative findings provided further context for these quantitative changes. Students described feeling less isolated, more emotionally supported by peers, and more aware of their personal strengths, which may help explain the reductions in psychological symptoms and changes in coping‐related awareness observed in the quantitative results.

Our qualitative findings showed that the psychoeducation program was generally positively perceived by the participants and found to be quite effective. In particular, the timing of the intervention and its interactive nature stood out as the main elements that increased the participants' motivation and interest in the program. Several studies in the literature have stressed that the timing of interventions following traumatic events is critical (Bonanno et al. 2010; Dyregrov and Regel 2012; Roberts et al. 2009). In addition, cultural relevance was highlighted by students and is frequently mentioned in the literature as an important factor in the acceptability of interventions (Bernal et al. 2009; Sue et al. 2009). For example, the use of relevant characters, such as Nobel Prize‐winning scientist Aziz Sancar, in addition to each contextual relevance, has a positive impact on self‐confidence and motivation among adolescents as shown in the literature (Niemiec 2012; Proctor et al. 2011; Shoshani and Slone 2013).

One of the benefits of this program is that it allows students to share their feelings and feel less lonely when they realize that others have the same fears and concerns. The program also facilitated social support among peers, which proved to be important for psychological recovery following traumas such as earthquakes (Hobfoll et al. 2007). Social support also has a role in stress management and improving mental health (Choi and Toma 2014; Thoits 2011). Additionally, studies showed that group‐based psychoeducation in post‐trauma interventions helps identify and challenge negative thoughts that contribute to mental health problems (Litwack et al. 2022; Sloan et al. 2012; Yalch et al. 2022). Rather than indicating the development of resilience as a stable trait, the findings suggest short‐term improvements in psychosocial resources such as emotional awareness, adaptive coping orientations, and social connectedness. These elements are considered key components of post‐disaster psychosocial adaptation rather than comprehensive resilience.

To improve the intervention, participants suggested reducing group size and including their families in the intervention process. They emphasized that including families in psychoeducation will enable both themselves and the family to cope better with the traumatic events in general. This aligns with the literature that highlighted the importance of family‐based interventions (Pfefferbaum and North 2020). Additionally, reducing group size enables greater active participation and more space for individual expression, and facilitates more effective learning and deeper interaction (Yalom and Leszcz 2005).

Beyond situating the findings within the existing literature, it is also important to consider their practical relevance for post‐disaster mental health practice. The study findings have several practical implications for mental health practitioners, disaster response teams, and policymakers working with adolescents in post‐disaster settings. The observed reductions in psychological symptoms and improvements in active coping indicate that school‐based psychoeducational programs grounded in positive psychology can serve as feasible and effective early psychosocial interventions after large‐scale disasters. The cultural relevance of the intervention content appears to have enhanced engagement, suggesting that similar programs can be adapted to different post‐disaster contexts by incorporating locally relevant examples and values. From a policy perspective, the brief, structured, and group‐based nature of the intervention supports its scalability within educational settings, making it a cost‐effective strategy for addressing adolescent mental health needs following disasters.

### 4.1 Limitations

This study has several limitations that should be considered when interpreting the findings. First, the sample comprised 74 students from a single school, limiting the generalizability of the results to other adolescent populations and post‐disaster contexts. Second, the use of a one‐group pretest–posttest design without randomization or a control group restricts causal inference, as observed changes cannot be conclusively attributed to the intervention. Third, the absence of follow‐up assessments precludes concluding the sustainability of the observed changes over time. Finally, the large group size during implementation may have limited individualized engagement, potentially affecting the depth of participant involvement. Despite these limitations, the pilot nature of the study provides valuable preliminary insights into the feasibility and short‐term psychosocial impact of school‐based positive psychology interventions in post‐disaster settings.

## 5 Conclusion and Recommendations

This study examined the impact of a four‐session positive psychology‐based psychoeducation program on the psychological symptoms, coping strategies, and future expectations of senior students in a village high school during the post‐earthquake period. Following the intervention, a significant decrease in the students' psychological symptoms (anxiety, depression, somatisation, hostility, and negative self‐perception) was noticed, indicating that the psychoeducation program contributed positively to psychological well‐being. Additionally, significant improvements were observed in active and negative coping, but no significant change in avoidant coping strategies. Furthermore, students developed more positive future expectations, especially regarding family and marriage‐themed sub‐dimensions.

Students' qualitative feedback supported the quantitative results, highlighting the intervention's positive impact on their well‐being. Students described the intervention as timely, as it coincided with the exam period after the earthquake. Students also highlighted the cultural relevance of the intervention, using examples from their context. To improve the intervention, students suggested involving their families and reducing group size to allow more interaction.

In conclusion, a psychoeducation program built on positive psychology principles contributed not only to the reduction of psychological symptoms but also to improving positive mental health, including recognizing personal strengths, developing positive relationships, and more conscious planning for the future.

Based on the findings of our research and the experiences of the students, we recommend the following:

- Psychological interventions following traumatic events should not be only symptom‐focused, but also should integrate positive psychology approaches that nurture individuals' strengths, sense of hope, and future expectations. These approaches can enhance post‐traumatic resilience and growth and contribute to positive mental health outcomes.
- Interventions should be relevant to the context and culture. Listening to participants' feedback and involving them in the adaptation process is key to improving the intervention's acceptability and effectiveness.
- Health interventions targeting children and adolescents should also include their families, as family plays an important role in children's emotional and social development.
- When conducting group interventions, smaller groups may be preferred to increase the level of participation and interaction among participants.
- Although the intervention showed short‐term benefits, its long‐term effects remain unknown; thus, 3–6 month follow‐up studies and randomized controlled trials are needed to assess sustained effectiveness.
- To ensure the scalability and sustainability of this intervention, we suggest using teachers as delivery agents. Teachers could be trained to deliver the intervention and reach more students.

## Author Contributions

**Veysel Kaplan:** conceptualization, investigation, funding acquisition, writing – original draft, methodology, validation, visualization, writing – review and editing, formal analysis, project administration, data curation, supervision, resources, software. **Mehmet Emin Düken:** writing – original draft, data curation, formal analysis. **Abanoub Riad:** formal analysis, supervision, writing – review and editing. **Muhammad Alkasaby:** investigation, writing – original draft, methodology, validation, visualization, writing – review and editing, formal analysis.

## Consent

Informed consent was obtained from all individual participants included in the study.

## Conflicts of Interest

The authors declare no conflicts of interest.

## Supporting information


Supporting File

## Data Availability

The data analyzed during the current study are available from the corresponding author on reasonable request.
