# Supplementary material for: Assessing the Impact of a Positive Psychology School‐Based Psychoeducation on the Mental Health of Adolescents Affected by the 2023 Earthquake in Türkiye
Source: J Adolesc. 2026 Apr 8;98(5):1454–64. doi: 10.1002/jad.70153 (PMC13338648; doi:10.1002/jad.70153)
Supplement: Supplementary file 1 — Supporting File [file JAD-98-1454-s001.docx]

**Details and Procedure of the Sessions**

The psychoeducational sessions were designed by integrating core principles from positive psychology and established post-disaster mental health frameworks. While the intervention does not represent a manualised disaster risk reduction programme, its components align with key elements commonly used in school-based psychosocial interventions following disasters, including strengths-based approaches, cognitive reframing, emotional regulation, and social support enhancement. The programme was adapted to the cultural and contextual characteristics of adolescents living in rural, earthquake-affected settings in Türkiye.

Specifically, the first session focusing on character strengths draws on strengths-based and meaning-oriented approaches used in positive psychotherapy and post-disaster psychosocial care. The second session on optimism and hope incorporates cognitive reframing techniques commonly applied in trauma-informed and CBT-based interventions. The third session integrates self-compassion and basic stress regulation strategies consistent with psychological first aid and emotional regulation frameworks. The final session on positive relationships reflects social support–oriented models emphasised in disaster recovery literature, which highlight interpersonal connectedness as a protective factor in post-disaster adaptation.

Although the intervention was not designed as a formal disaster risk reduction programme, it addresses psychosocial risk factors following disasters by strengthening individual and relational resources shown to support post-disaster adaptation among adolescents.

**Session 1 (Character Strengths and Virtues):** This session started with a general introduction and explanation of the intervention’s aims. Students were given information to raise awareness about the strengths and positive characteristics that exist in every individual, which is the main point of the session. After that, we opened an interactive discussion with students based on the following questions:

- Do you have idols/people you like very much around you?
- Which characteristics of these people do you like and why?
- Do you have characteristics that you like in yourself? Why?
- What are your behaviours that make people around you happy?

After that, we asked participants to choose pairs and state the good qualities they like about each other. The aim of this activity is to increase the individual's self-awareness and self-worth by receiving feedback from others. Finally, the participants discussed what they could do to improve the society/country they live in. In general, this session was built on Positive Psychotherapy's dynamics of growing together as a part of society and being happy while making others happy.

**Session 2 (Optimism and Hope for the Future):** This session focused on enabling participants to be optimistic in their daily lives and to have positive future expectations. Participants were given some situations or scenarios of issues they encounter on a daily basis and were asked to reflect on these scenarios. Scenarios focused on being able to draw possible better/not worse/alternative conclusions from specific situations. Reframing was done on situations that adolescents may encounter in their daily lives. Questions discussed include the following:

- What does a friend who passes by without greeting us make us think about?
- If the stated thought is negative, what could be the alternative to the first thought that comes to mind
- How do we feel about the exam on which we received a bad grade?
- If the stated thought is negative, what could be the alternative feeling?

The aim here is for adolescents to develop alternative feelings and thoughts to challenge the negative thoughts in the situations given. Afterwards, the discussion moved to future expectations by posing the following questions:

- Does dreaming feel good?
- What do I want to be/do in the future?
- What is the importance of the details of our dreams?
- How can we make our dreams real?

The advantages of positive thinking were also mentioned in this session. Participants were taught the positive interpretation technique of Positive Psychotherapy in order to use events/situations/people as a trigger for their personal development, and the session was structured around this technique. Then, the stories of well-known, successful people from Turkey and the world were presented. We presented how those popular figures progressed in their careers, starting from their childhood years, and the struggles they experienced. At the end, participants were asked to write what they wanted to be/where they wanted to be in the future in the dream envelopes given to them and hang them on the branches of the trees in the school garden. The need to strive for these dreams, hung on the tree branches to grow like fruits, was discussed. The participants were also given notebooks. They were asked to make a checklist in this notebook about what they needed to do to realize their dreams. We explained that the steps written in this checklist resemble the light, heat and water needed to grow fruits.

**Session 3 (Self-Understanding and Struggle):** This session focused on participants being able to better understand themselves. We facilitated a discussion with participants around the following questions:

- What is making a mistake?
- Why do people make mistakes?
- How do you support people around you when they make mistakes?
- How do you feel when you make a mistake? What do you say to yourself?

After the discussions, information was provided on the fact that problems, setbacks or mistakes are a part of life and that mistakes can be a motivation for improvement. The aim was to increase students’ self-esteem by allowing them to be at peace instead of getting angry with themselves after a mistake they made. The remaining time of the session focused on developing skills to cope with difficult situations. We facilitated another discussion about what students do when they feel angry, under pressure, or worried. After that, participants were taught breathing and muscle contraction-relaxation exercises accompanied by music in the school garden. Then, students were asked to sit around a tree they chose with the group of students they chose and practice the techniques taught while interacting with each other. The fact that participants lived in rural areas was a significant factor in choosing this method. Since trees are quite abundant in their physical environment, children will be able to apply the coping techniques taught around a tree when they feel stressed, angry, or anxious.

**Session 4 (Positive Relationships):** This session aims to help participants gain skills to develop positive relationships. We facilitated an interactive discussion around the following questions:

- How do you evaluate your relationships with your environment?
- What are the general characteristics of people you love very much or who are loved by everyone?
- What can be said to make people feel good?
- What communication methods can be used to start and maintain positive relationships?

Following these discussions, information was provided about the effects of our relationships on us and those around us. Topics such as healthy relationships, you-me language, and effective communication skills were discussed. Empathy was also discussed, and the importance of trying to understand how others feel when faced with challenging life events was mentioned. Discussion was supported by giving examples of situations adolescents may frequently encounter in their lives, such as:

- How do you approach a friend who did poorly on an exam when you feel very sad?
- How do you approach your best friend when you feel anxious because he/she moved to another city?
- How do you approach a friend when he/she tells you that he/she is having conflict with his/her family and that he/she feels that they do not love him/her?

The session ended with these discussions. Then, in an extra 15 minutes, a closing was made in which mutual good wishes and thanks were given. Some students wanted to take photos. The school administration made a closure speech, and the psychoeducation ended.
